# Toward bidirectional FHIR–OMOP CDM transformations using TermX to support the secondary use of real-world health data within a patient-centered digital health paradigm

**DOI:** 10.3389/fmed.2026.1736785

**Published:** 2026-02-12

**Authors:** Hanna Kätlin Ardel, Rainer Randmaa, Igor Bossenko, Gunnar Piho, Peeter Ross

**Affiliations:** 1 Department of Software Science, Tallinn University of Technology (TalTech), Tallinn, Estonia; 2 Department of Health Technologies, TalTech, Tallinn, Estonia; 3 Research Department, East Tallinn Central Hospital, Tallinn, Estonia

**Keywords:** bidirectional transformations, European Health Data Space (EHDS), Fast Healthcare Interoperability Resources (FHIR), health data interoperability, Observational Medical Outcomes Partnership (OMOP) Common Data Model (CDM), secondary use of health data, terminology and data interoperability tool TermX

## Abstract

The increasing digitization of healthcare has led to vast amounts of clinical data, much of which remains underutilized for research. While Health Level Seven (HL7) Fast Healthcare Interoperability Resources (FHIR) improves interoperability in clinical care, it's primarily designed for real-time data exchange to support diagnosis and treatment, rather than for secondary use of health data. As a result, transforming FHIR data into standardized models such as the Observational Medical Outcomes Partnership (OMOP) Common Data Model (CDM) remains a challenge. This study employs TermX, an open-source terminology and data interoperability platform designed to enhance health data interoperability and support knowledge management. This allowed us to create bidirectional transformation rules between FHIR and OMOP CDM. Using the Design Science methodology, we developed and validated a set of standardized transformation rules that support bidirectional mapping of vital signs data between FHIR and OMOP CDM. In these transformations we used synthetical FHIR JSON data, focusing on five main resources—*Observation, Patient, Encounter, Organization*, and *Practitioner*. The focus of this work is primarily on methodological mapping rather than processing real-world datasets; the evaluation concentrates on mapping coverage, i.e., the proportion of FHIR elements that can be reliably transformed into OMOP CDM structures and vice versa. The resulting rules achieved 74% mapping coverage from FHIR to OMOP CDM tables, with unmapped elements primarily related to structural discrepancies. Mapping from OMOP CDM to FHIR reached approximately 23% coverage, capturing mostly values that were previously mapped from FHIR to OMOP CDM. These percentages reflect variations in the standards' structure and granularity. The application of TermX shows the feasibility of reusable, standards-based transformations that support the secondary use of real-world clinical data for medical research and analysis. By addressing key technical and semantic interoperability challenges, this work contributes to advancing digital health interoperability and supports the objectives of the European Health Data Space.

## 1 Introduction

Health data collected during clinical care make up a large part of the global data landscape (1, 2). Today, most health data is not being reused (e.g., 97% of all generated hospital data remains unused (3–5). This means that most clinical data is only being used for primary use—for treatment, limiting its potential use in medical research toward improving healthcare outcomes and operational efficiency (6).

Secondary use of health data offers substantial benefits for quality, cost efficiency, research, and public health (7, 8). It supports evidence-based decision-making, fosters innovation, advances research, and strengthens public health initiatives (5, 8). Already, a report published in 2009 by PricewaterhouseCoopers, “Transforming Healthcare through Secondary Use of Health Data” (7), emphasized that utilizing clinical data in this way can enhance treatment outcomes and reduce costs by enabling the discovery of disease patterns, identification of new treatment options, and more efficient resource allocation (8).

The problem addressed in this paper is highly relevant in the context of the European Health Data Space (EHDS). The EHDS is grounded in European Union (EU) regulations and aims to enable the secure and efficient interoperability of health data across the healthcare systems of all EU Member States (9). Its goal is to create a unified framework that allows individuals to easily access and control their electronic health data, while also facilitating the trustworthy and safe use of such data for research, innovation, and data-driven policymaking (9).

Within the EHDS framework, particular emphasis is placed on data quality, standardization, and security. Among the key interoperability frameworks, HL7 FHIR plays a central role as the standard for exchanging clinical information, while the OMOP CDM serves as a reference standard for structuring data for secondary use (10).

The paper is organized as follows: Section 2 defines the problem, and Section 3 outlines the methods used for creating the transformation rules between HL7 FHIR and OMOP CDM. Section 4 presents the results—a set of standardized transformation rules. Section 5 discusses the evaluation, Section 6 addresses implications, and Section 7 concludes with suggestions for future work.

### 1.1 Significance and contribution

This paper addresses a broader challenge in digital health: most clinical data collected today cannot be reused for research because the systems storing them are not designed for secondary use (11), even though it could inform better treatments, support innovation, and improve public health.

By creating standardized, reusable transformation rules between HL7 FHIR and the OMOP CDM, this paper contributes empirical insights into the accuracy, data loss, usability, and interoperability constraints. These findings highlight which aspects of clinical data can be reliably reused for research and which limitations arise from structural and semantic differences between the standards, independent of any specific transformation tool.

The transformation rules developed in this work demonstrate a practical pathway for converting everyday clinical data into research-ready formats. This supports the secondary use of existing health records and improves the quality and comparability of real-world evidence. In doing so, the paper contributes to the goals of the European Health Data Space by improving interoperability and enabling more effective secondary use of health data for research, innovation, and public health.

Specifically, this paper contributes (i) generalized, executable, and reusable rule-based transformation patterns between HL7 FHIR and OMOP CDM, implemented using the FHIR Mapping Language (FML) and integrated with ConceptMap-based terminology mappings; (ii) the demonstration of transformation logic on a concrete use case in both directions, including one-to-one and one-to-many/many-to-one mappings; and (iii) a systematic analysis of technical and semantic limitations, including terminology mismatches, key management challenges, and data loss arising from structural and granularity differences. In addition, the paper provides a measurable assessment of mapping coverage and derives conclusions applicable to rule-based transformation frameworks beyond the specific tooling used, with TermX serving primarily as an execution environment.

## 2 Problem statement

Today, healthcare data analysis has struggled to fully utilize real-world clinical data for research (4, 12–14). Most healthcare data is primarily collected to support patient care, rather than for secondary use purposes such as research (15). HL7 FHIR is primarily designed for the real-time exchange of health information between different systems (16), but not with a purpose for the long-term storage or secondary use of clinical data (17, 18). Whereas the OMOP CDM is designed specifically for secondary data use—it enables the representation of observational health data in a standardized format suitable for data analysis and research (17, 19).

Transforming HL7 FHIR data to OMOP CDM table structures provides an opportunity to transform clinical data into an analysis-ready format (20). Conversely, transforming OMOP CDM data back to FHIR can support selected secondary use cases, but the resulting FHIR resources are generally not suitable for direct use in clinical workflows due to missing mandatory fields and OMOP CDM de-identification logic. The reverse transformation is beneficial, in the longer term, to get data from research, questionnaires, or medical examination back to clinical data that's connected to the patient and are reusable for the physician. This would create bidirectional data flow: data collected from clinical data to research, and data used and collected in research back to the patient's health data.

Transforming FHIR data into research-ready formats such as the OMOP CDM remains a complex task, as the standards differ in purpose, structure, and intended use (21, 22). Barriers can be broadly categorized into technical challenges (e.g., differences in data structures, aligning mandatory and optional fields, divergent standard purposes) and semantic interoperability challenges (e.g., compatibility across coding systems, aligning data types and terminology). Most existing solutions have been developed in a project-specific manner, and only a few studies have shared their ETL (*Extract, Transform, Load*) processes (22).

This paper addresses interoperability challenges between HL7 FHIR and the OMOP Common Data Model by proposing a rule-based and reusable mapping methodology for defining transformations in both directions between HL7 FHIR and OMOP CDM. The approach emphasizes reusability, executability, and validation, moving beyond descriptive mapping guidance toward reusable transformation rules that can be directly integrated into ETL processes. The methodology is implemented and evaluated using TermX, a knowledge management and semantic interoperability platform (23–26), which supports standardized access to terminologies, data models, and schemas. In this paper, TermX serves as the implementation environment that enables the definition, reuse, and validation of the proposed transformation rules and terminology mappings.

The approach focuses on core FHIR resources—Observation, Patient, Encounter, Practitioner, and Organization—and mapping them to corresponding OMOP CDM tables: Measurement, Person, Visit Occurrence, Provider, and Care Site. Aligning the semantic layers of both standards, particularly in their use of terminologies, is a part of this process. A generalized, reusable transformation approach can reduce data standardization efforts, simplify secondary use workflows, and improve data accessibility for research.

## 3 Research questions

This paper addresses how FHIR-formatted clinical data can be transformed into the OMOP CDM for research use with a focus on technical and semantic interoperability. The objective is to establish a reliable, replicable, and reusable process for transformation between HL7 FHIR and OMOP CDM.

The research is guided by two key questions:

- What are the accuracy, data loss, and usability characteristics of the transformations between HL7 FHIR and OMOP CDM?
- What are the key technical and semantic interoperability challenges in bidirectional transformations between FHIR and OMOP CDM?

## 4 Methods

This paper applies the Design Science (DS) methodology, which supports the systematic creation and evaluation of IT artifacts addressing real-world problems (27).

Following the Design Science Research methodology, this paper addresses the three main phases of the design cycle: (1) problem identification, which defines the interoperability challenges motivating the paper; (2) treatment design, which results in the creation of a reusable transformation artifact; and (3) treatment validation, which evaluates the artifact with respect to predefined structural and semantic criteria. The transformations are refined iteratively. After each testing cycle, identified issues are corrected and re-tested, iteratively refining the transformation rules based on validation feedback. Each phase is described in the following subsections.

In this paper, the artifact consists of a set of reusable transformation rules between HL7 FHIR resources and OMOP CDM table structures, implemented in the TermX platform (28, 29). A *transformation* refers to the overall process of transforming data from one format or structure to another (28, 29), while maintaining original semantic meaning. A *transformation rule* is a specific instruction or set of methods that defines how a particular piece of data should be transformed (28, 29). A *transformation component* is a visual representation of a transformation rule in the TermX Visual Editor, containing FML code that implements the required transformations(28, 29). These components constitute the implemented design artifact of this paper.

### 4.1 Research toward reusable transformation components

This subsection describes the application of the Design Science cycle to the development of reusable transformation components.

#### 4.1.1 Problem identification

Secondary use of health data offers significant potential, but remains limited, as healthcare systems have primarily been designed around the administrative and financial needs of primary care delivery (11). As a result, integrating data for secondary purposes has proven complex. The main hindering factors in Estonia include the lack of cross-sectoral regulation and coordination, as well as limitations related to data standardization (30).

In Estonia, health data exchange continues to rely mainly on document-based formats, particularly the HL7 Clinical Document Architecture (CDA) standard (31), where data are shared as XML files (32). To address this, a national decision was made in 2020 to modernize the health information system and adopt HL7 FHIR as the primary standard for clinical data exchange (33).

Previous research has focused on achieving semantic interoperability between HL7 CDA and HL7 FHIR (28), introducing methods for creating and validating reusable, visual transformation components. Building on that foundation, the present paper extends the interoperability pipeline from primary data exchange (CDA to FHIR) to secondary data use (FHIR to OMOP CDM), establishing a reusable approach for transforming FHIR-formatted data into the OMOP CDM.

#### 4.1.2 Treatment design

The treatment design phase operationalizes the identified interoperability challenges into a concrete design artifact in the form of reusable transformation components. The design objective was to develop reusable transformation components linking HL7 FHIR resources to OMOP CDM tables. Components were implemented in TermX, following the HL7 Vulcan Implementation Guide (20) and OMOP CDM specifications (34).

The design aimed to ensure semantic compatibility between the standards and to support future implementation of ETL processes in compliance with the physical OMOP CDM data model. Link tables are defined to associate FHIR resource identifiers with anonymized OMOP CDM identifiers, aligning with OMOP CDM's de-identification principles.

Depending on the transformation direction, the logical and physical OMOP CDM table structures are defined in TermX and used as target or source tables. The platform's connection to an HL7 FHIR server enabled direct access to FHIR resource structures, eliminating the need for manual schema definition. Transformations are created using TermX's graphical FHIR Mapping Language (FML) editor, mapping the core FHIR resources—*Observation, Patient, Encounter, Organization*, and *Practitioner*—to the corresponding OMOP CDM tables: *Measurement, Person, Visit Occurrence, Care Site*, and *Provider*. These mappings are bidirectional.

Terminology mappings between HL7 and OHDSI vocabularies are first drafted manually and later imported into TermX's concept mapping module to establish semantic relationships. Technical and semantic issues identified during development are iteratively documented and resolved.

To demonstrate the approach, vital signs are selected as a use case for transformation. Vital signs are key physiological indicators, such as respiratory rate, heart rate, body temperature, oxygen saturation, weight, height, head circumference, body mass index, and blood pressure (35, 36). They are essential in both clinical decision-making and research, and their systematic analysis has been shown to improve the early detection of clinical deterioration (36, 37).

**Figure 1:**
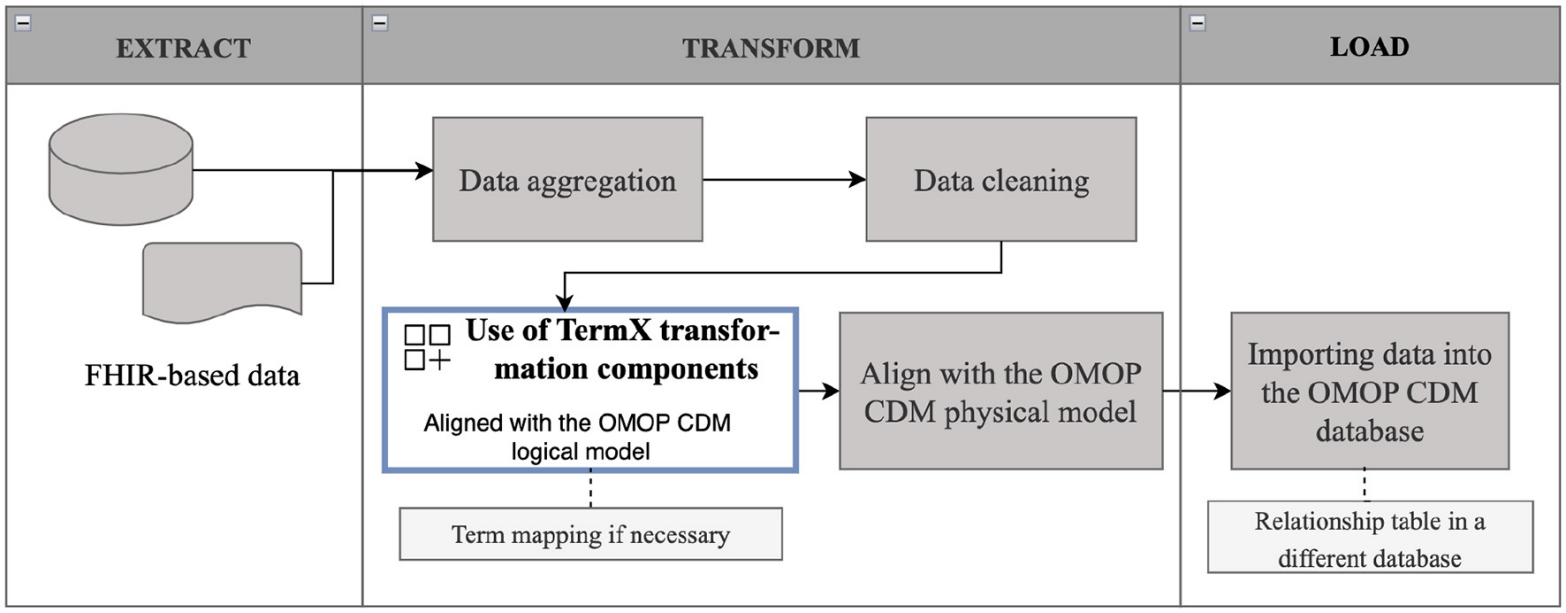
ETL process with TermX transformation components.

Figure 1 illustrates the developed transformation components' role in the overall ETL process for transforming data from HL7 FHIR to OMOP CDM.

#### 4.1.3 Treatment validation

Following the Design Science validity framework, the study advances a *criterion claim* as the primary contribution, which is sufficient for early-stage and novel artifacts (38).

The prototype achieved Technology Readiness Level (TRL) 3, corresponding to proof-of-concept validation (39). Transformation correctness was validated manually using the built-in Transform function of TermX. At higher TRL levels, validation should additionally include execution of the FML scripts in independent environments and verification using alternative tooling to ensure tool-independent correctness and reproducibility. Future work will therefore focus on validation in controlled environments and large-scale evaluation using real-world clinical datasets and complete ETL pipelines, including the integration of the transformation rules into operational data flows.

Testing first verified the structural correspondence between HL7 FHIR resources and OMOP CDM table structures, followed by transformation tests using vital signs data. This paper uses example HL7 FHIR resources in JSON format, specifically synthetic *Observation, Organization, Practitioner, Patient*, and *Encounter* resources representing vital sign measurements (body temperature and blood pressure). The example instances were sourced from (40), following the HL7 FHIR specifications (16). OMOP CDM test data are mostly constructed manually, based on the corresponding physical data model table structures, using the transformed FHIR data as a reference. Multiple scenarios are executed to evaluate the handling of incomplete or incorrect input fields and to verify the correctness of terminology mappings.

Validation addressed two main dimensions:

- Structural consistency: verifying that transformed data met target-structure requirements (data types, mandatory fields, and constant values where applicable).
- Semantic accuracy: confirming that transformed elements preserved their intended meaning and that terminology mappings are valid within the target standard.

### 4.2 Implementation environment: TermX platform

In this paper, TermX is used as an implementation platform for defining and testing transformation rules between HL7 FHIR and OMOP CDM. TermX is an open-source knowledge management and collaboration platform developed as part of a doctoral project at Tallinn University of Technology (TalTech) (24, 26). The platform is designed to strengthen semantic interoperability and to facilitate standardized data exchange between systems.

TermX integrates a terminology server, a wiki environment, tools for creating and publishing resources, and a graphical editor for defining FHIR Mapping Language (FML) transformations (25). The platform supports terminology alignment, data-model design, and data transformation between different standards. In addition, it provides access to published terminologies and schemas, ensuring compatibility with the FHIR standard and other international specifications (24).

The central component of TermX is a graphical FML editor, which enables the creation and management of rule-based transformations between HL7 FHIR structures and models such as the OMOP CDM (41). The platform also supports the definition of reverse transformations. FML itself is a transformation language defined in the official FHIR specification and is based on FHIRPath, the formal query language of FHIR (42). FML leverages FHIRPath functions to specify the conditions, values, and structures of transformation rules.

TermX supports data transformation across heterogeneous data models through a graphical user interface. Users can visually create, test, and manage mapping rules, which are automatically compiled into executable FML scripts. These scripts can be deployed on a FHIR server to perform the defined transformations (25). This approach enables the definition and testing of transformation logic without requiring detailed knowledge of FML syntax.

TermX is used as the implementation environment; the transformation logic, validation criteria, and observed limitations described in this work apply to other rule-based transformation frameworks that support HL7 FHIR and OMOP CDM.

## 5 Transformation of vital signs data

This section discusses mapping vital signs from the HL7 FHIR standard to the OMOP CDM. We describe the overall transformation process but focus in detail on the transformation of the Measurement table from FHIR to OMOP CDM, and the bidirectional mapping of vital signs data, as these represent the core components of the use case. Other OMOP CDM tables involved in the process are not discussed in depth.

The following subsections demonstrate how the transformation rules, developed and validated as described in Section 4, are applied to the vital signs use case.

### 5.1 Mapping FHIR resources and OMOP CDM tables

The following subsection describes how elements within these resources are mapped to the corresponding OMOP CDM table fields using TermX.

To define transformation rules from FHIR data to OMOP CDM table structures, we first analyzed the relevant OMOP CDM tables. Vital signs data in FHIR is represented in the *Observation* resource, which corresponds to the *Measurement* table in OMOP CDM.

**Figure 2:**
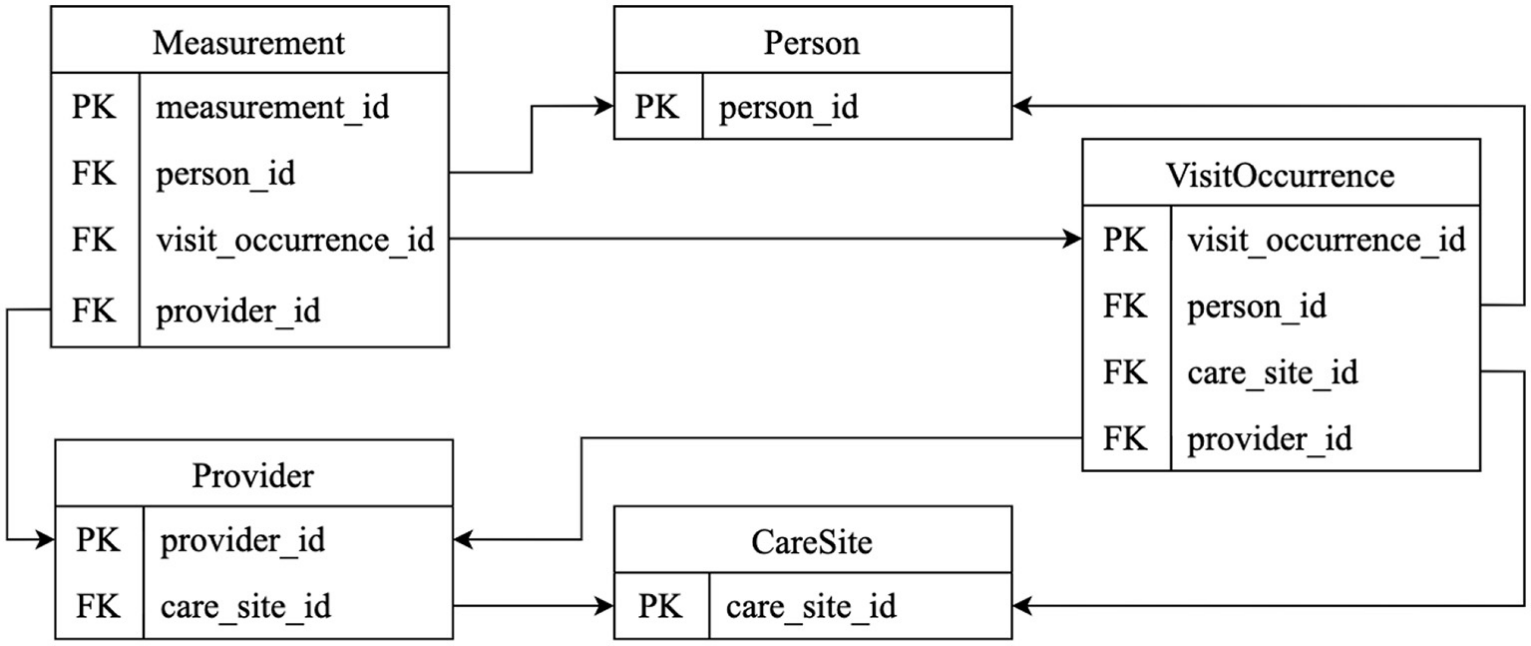
OMOP CDM tables used in vital signs transformation.

As a result, we established transformation rules for six OMOP CDM tables related to vital signs. Figure 2 illustrates the relationships between the tables used in this transformation. As shown in Figure 2, OMOP CDM maintains data integrity through foreign keys linking related tables, a structure that is preserved during the transformation process.

Establishing these correspondences defines the structural backbone for transforming FHIR formatted clinical data into an analysis-ready OMOP CDM dataset.

Table 1 presents FHIR resources that are used for transforming data to a specific OMOP CDM table structure. In the vital sign transformation context, these mappings can also be applied in the reverse direction.

**Table 1:** FHIR resources that are used as sources for OMOP CDM tables.

**HL7 FHIR resource**	**OMOP CDM table**
Organization	Care Site
Practitioner	Provider
Patient	Person
Encounter	Visit Occurrence
Observation	Measurement

### 5.2 Transformation process in TermX

This subsection demonstrates the transformation process in TermX, using the OMOP CDM Measurement table as the primary example. Because vital signs data are represented as FHIR Observation resources, which correspond directly to the OMOP CDM Measurement table, this example illustrates the core logic of the FHIR-to-OMOP transformation.

#### 5.2.1 The transformation of FHIR observation to OMOP CDM measurement

The OMOP CDM *Measurement* table stores results obtained from the systematic examination or testing of a person or their specimen. These include, for example, vital signs, laboratory results, and other quantitative findings (34). Each record in the table represents a specific measurement result, expressed either as a numerical value (e.g., respiratory rate, blood glucose level) or as a categorical value (e.g., *positive, negative*).

In HL7 FHIR, such results are primarily represented using the *Observation* resource. This resource is used not only for measurements and test results, but also for tracking diagnoses, monitoring the course of disease, and identifying patterns or demographic changes (16). As one of the core resources for documenting healthcare, the *Observation* resource includes a wide range of data: vital signs, laboratory test results, and measurements captured by medical devices.

Compared to OMOP CDM, the FHIR *Observation* resource is broader and more flexible. It accommodates both quantitative measurement results and qualitative clinical observations (16). In OMOP CDM, these types of information are split across two different tables (34):

- Quantitative measurements are stored in the *Measurement* table.
- More general or qualitative clinical observations are stored in the *Observation* table.

This conceptual difference is central to defining transformation rules. While many FHIR *Observation* instances map directly to the OMOP *Measurement* table, others must be redirected to the OMOP CDM *Observation* table depending on their content and type.

To systematically define the transformation logic, each element of the HL7 FHIR Observation resource is examined for correspondence with fields in the OMOP CDM Measurement table. Table 2 summarizes the transformation outcomes for Observation resource elements, distinguishing between direct transformations, indirect transformations that require terminology mapping or foreign key resolution, and elements that are not represented in the OMOP CDM Measurement table.

**Table 2:** FHIR observation resource elements compared to OMOP CDM measurement table fields.

**FHIR resource element**	**OMOP CDM table field**	**Transfor-mation**	**Comment**
Identifier	Measurement_source_value	Indirect	This value is used in the primary key creation, but the data is not saved in the OMOP CDM database
Instantiates[X]	–	–	–
Based On	–	–	–
Triggered By	–	–	–
Part Of	–	–	–
Status	–	–	–
Category	–	–	–
Code	Measurement_concept_id	Indirect	Concepts are transformed through concept maps
Subject	Person_id	Indirect	Foreign key value is transformed directly; the reference type is set as a constant to define the foreign key target
Focus	–	–	–
Encounter	Visit_occurrence_id	Indirect	Foreign key value is transformed directly; the reference type is set as a constant to define the foreign key target
Effective[X]	Measurement_date, measurement_datetime, measurement_time	Indirect	In OMOP CDM the time of measurement is represented through separate fields - date, time, and combined date-time values
Issued	–	–	–
Performer	Provider_id	Indirect	Foreign key value is transformed directly; the reference type is set as a constant to define the foreign key target
Value[X]	Operator_concept_id, value_as_number, unit_source_value, unit_concept_id, value_source_value	Direct, indirect	Fields that contain concepts are transformed through terms mapping; other values are directly transformed
Data Absent Reason	–	–	–
Interpretation	–	–	–
Note	–	–	–
Body Site	–	–	–
Body Structure	–	–	–
Method	–	–	–
Specimen	–	–	–
Device	–	–	–
Reference Range	Range_low, range_high	Direct	Initial values are directly transformable using the same data types
Has Member	–	–	–
Derived Form	–	–	–
Component	–	–	–

The Transformation column classifies the type of transformation applied, while the Comment column provides additional explanation of the applied mapping logic. Elements marked as not transformable indicate cases where the OMOP CDM Measurement table does not contain a corresponding field for the given FHIR element.

As shown in Table 2, only a subset of FHIR Observation elements have direct equivalents in OMOP CDM, reflecting the different purposes of the two standards.

To transform the OMOP CDM table from a FHIR resource, we compared the input data to the output and *vice versa*. The FHIR resource structure is more detailed to ensure primary care quality. All this, for example, data to identify a person, is not necessary for secondary data usage. Because of that, in the transformation process, some of the data will be lost and which makes it more difficult to later transform the same OMOP CDM table to the FHIR resource.

This loss of detail illustrates a fundamental trade-off between clinical documentation richness and analytical standardization in secondary data use.

**Figure 3:**
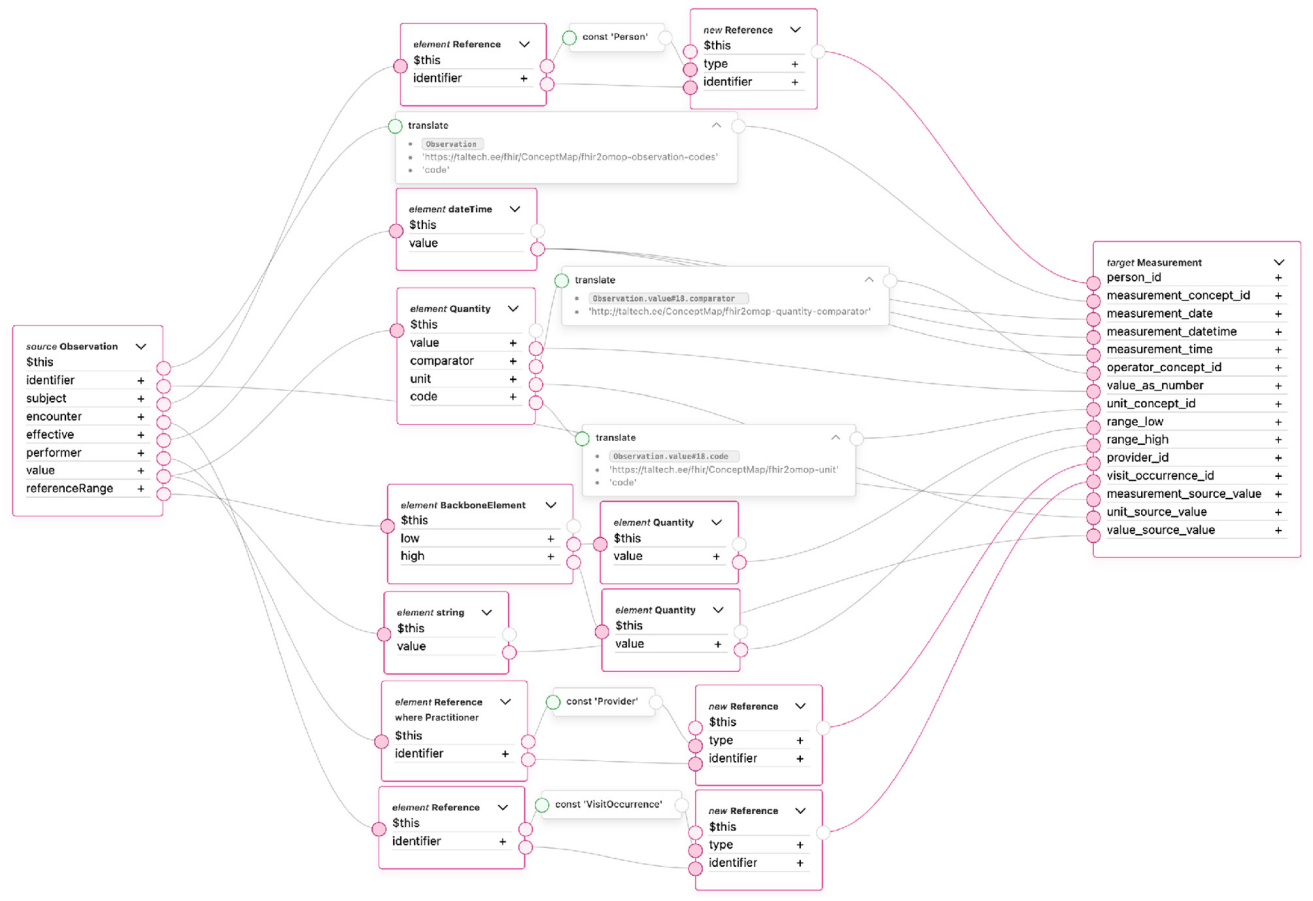
FHIR observation resource transformation to the measurement table in the TermX platform.

Figure 3 illustrates the mapping logic used to transform FHIR Observation resources into the OMOP CDM Measurement table. Each link in the figure represents a transformation rule defined using the FHIR Mapping Language (FML) within the TermX platform.

Measurement table core attributes—such as identifiers, codes, subject (patient), encounter, effective date/time, performer (provider), and measurement values—can be mapped directly. Reference ranges are represented through the range_low and range_high fields. However, many other elements of the FHIR Observation resource lack a corresponding representation in the OMOP CDM Measurement table and are therefore omitted.

The transformation establishes correspondences between key FHIR elements—such as subject, code, effectiveDateTime, valueQuantity, and referenceRange—and the respective OMOP CDM Measurement fields, including person_id, measurement_concept_id, measurement_date, value_as_number, range_low, and range_high. Each FHIR resource is linked to its corresponding OMOP CDM record using pseudonymous identifiers generated during the transformation process.

Because the Measurement table can contain both numerical and categorical results, the transformation logic depends on the value type. In the vital signs use case, all values are numerical; therefore, the value_as_number column is populated, while categorical fields such as value_as_concept_id and value_source_value are not used.

Mappings involving standardized terminologies—such as measurement_concept_id, operator_concept_id, and unit_concept_id—rely on predefined concept mapping tables. These mappings are referenced in the FML rules through the Translate function, which connects FHIR codes (e.g., LOINC) to equivalent OHDSI concepts (43). At the time of implementation, the Translate function is still under development; therefore, concept-based fields are not fully populated, which limited complete semantic testing.

Other OMOP CDM columns, including measurement_event_id and meas_event_field_concept_id are designed to link related measurement records. Because each Observation in this transformation represents a single measurement, these linking fields are intentionally left empty.

Overall, the mapping demonstrated that a single FHIR Observation resource can be systematically transformed into OMOP Measurement records while preserving both semantic meaning and structural consistency. This result confirms the feasibility of using TermX for rule-based, standards-compliant data transformations and establishes a reusable pattern for future extensions to other FHIR resource types. The following summarizes the core findings and implications of this transformation.

### 5.3 Terminology mapping in TermX

Terminology mapping plays a crucial role in ensuring semantic interoperability between HL7 FHIR and OMOP CDM. It enables the consistent interpretation of coded clinical data, allowing transformed datasets to retain their original meaning across standards.

During the transformation process, terminology mappings between OHDSI and HL7 terminologies are created as needed. When constructing concept mapping tables, the FHIR ConceptMap resource is used, which enabled the mapping of concepts during transformation without requiring the creation of separate OMOP CDM Vocabulary tables. In TermX, these concept mapping tables are maintained as reusable, rule-based components that can also be applied in other projects or transformations. Furthermore, during the ETL process, corresponding records for the OMOP CDM Vocabulary tables can be derived from these concept mapping tables.

Once the concept mapping tables are established, terminology alignment is performed based on the semantic meaning of the terms. When necessary, the context of use and accompanying documentation of the mapped terms are analyzed to ensure their correct correspondence to OMOP CDM terminology concepts.

**Figure 4:**
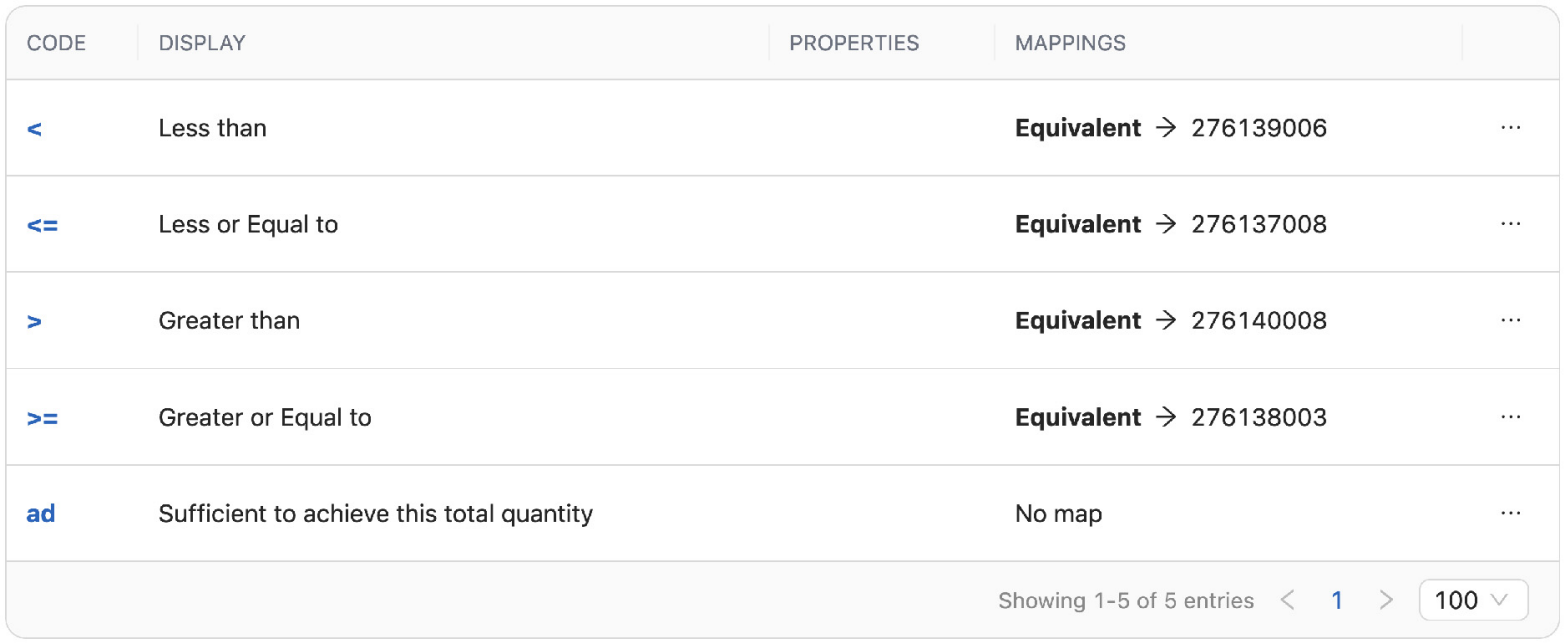
Mapping of HL7 and OHDSI terminology concepts using ConceptMap in the TermX.

An example of terminology mapping between HL7 and OHDSI terminologies is shown in Figure 4. This particular mapping is created for the operator_concept_id column in the OMOP CDM Measurement table and includes various comparison operators along with their corresponding OHDSI terminology identifiers.

While the implemented mappings covered all standard terms required for the transformations from FHIR to OMOP CDM, a complete bidirectional mapping of terminology is beyond the main scope of this work. This limitation is due to the extensive effort required for terminology alignment and the need for domain-specific clinical expertise. Moreover, a full mapping of OHDSI terminology to HL7 terminology is not always feasible, as not all OHDSI terms, especially non-standard concepts, are represented in HL7 terminologies.

Therefore, mappings for non-standard terms and reverse transformations are created only partially, with the primary goal of demonstrating the principles and methodology of terminology alignment. Despite these limitations, the approach demonstrated the feasibility of integrating terminology mapping directly into the transformation workflow within TermX, providing a scalable foundation for future, domain-specific expansion.

### 5.4 Vital signs transformation from FHIR to OMOP CDM

Vital signs are fundamental indicators of patient health, and their transformation provides a representative test case for evaluating the interoperability between HL7 FHIR and OMOP CDM. The transformation of vital signs data can be divided into two categories: (1) single-component vital signs, such as temperature or heart rate, and (2) composite measurements, such as blood pressure.

#### 5.4.1 Transforming body temperature, respiratory rate, heart rate, blood oxygen saturation, weight, height, head circumference, and body mass index data

For all single-component vital signs, the transformation process followed the same structure as the general Measurement table transformation. Each FHIR Observation resource is mapped directly to one OMOP CDM Measurement record. These vital signs—including respiratory rate, heart rate, body temperature, oxygen saturation, weight, height, head circumference, and body mass index—exhibit a one-to-one correspondence between FHIR and OMOP CDM representations. As such, their transformation is straightforward and served to validate the general transformation approach described earlier.

#### 5.4.2 Transforming diastolic and systolic blood pressure data

Among the vital signs, blood pressure transformation differs from other vital sign transformations in that systolic and diastolic blood pressure results are stored as two separate records in the OMOP CDM Measurement table. Therefore, a single FHIR Observation resource containing both measurements must be transformed into two OMOP Measurement records.

Due to FML limitations—which allow only one input and one output structure per transformation—a two-step transformation is implemented. In each step, a different transformation rule is applied to distinguish between systolic and diastolic blood pressure results based on their LOINC codes. LOINC serves as the terminology specified within the FHIR Observation resource:

1. Diastolic blood pressure transformation uses LOINC code 8462-4.
2. Systolic blood pressure transformation uses LOINC code 8480-6.

**Figure 5:**
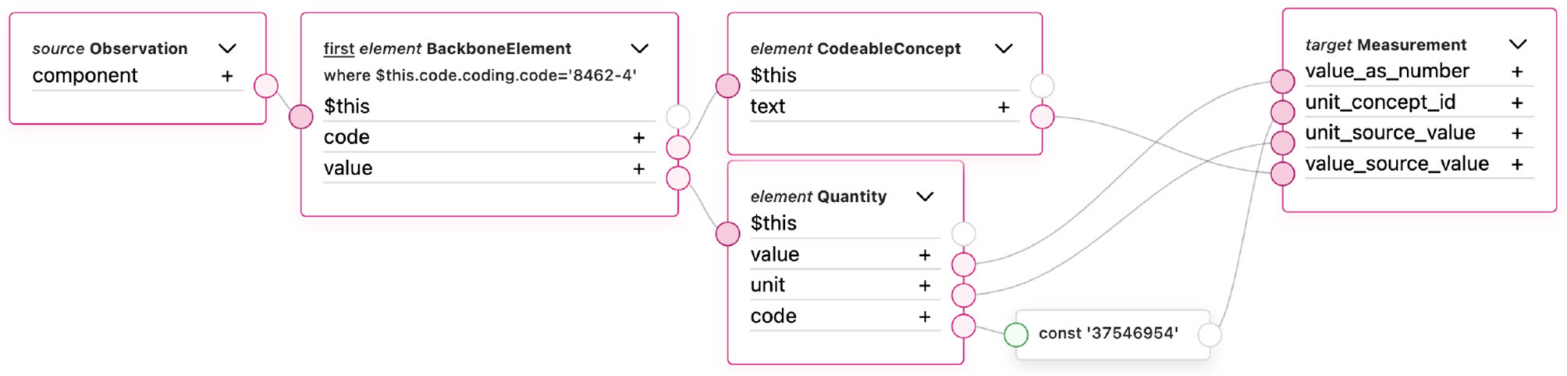
Transformation of the diastolic blood pressure value from the HL7 FHIR observation component structure in TermX.

Figure 5 illustrates the extraction of the diastolic blood pressure measurement result. Within the FHIR Observation resource's Component structure, where blood pressure values are stored, a conditional filter identifies the diastolic measurement based on its corresponding LOINC code. This value is then transformed into the value_as_number column of the OMOP CDM Measurement table. The measurement unit is stored in unit_source_value, and a textual description (in this case, “Diastolic Blood Pressure”) is stored in value_source_value. Additionally, the unit term can be defined as a constant or translated using the Translator function, as blood pressure units are always the same. The same logic is applied for systolic blood pressure transformation.

In addition to the above, OMOP CDM requirements specify that each transformed record must have a unique primary key and a link between related records. Primary key generation in this transformation follows the same approach as in other transformations described in this work. To prevent key uniqueness conflicts in later ETL process stages, the measurement_event_id column is populated with the resource identifier. This approach enables related Measurement records to be linked and uniquely identified through the combination of measurement_id, measurement_concept_id, and measurement_event_id.

In the subsequent ETL process, the measurement_event_id must serve as a foreign key linking the two blood pressure records. However, in the current implementation, this linkage cannot be created automatically because the two transformations are executed independently. Therefore, the meas_event_field_concept_id column is assigned the OHDSI standard terminology value 1147138, which indicates that the measurement_event_id key originates from the measurement_id column (43).

**Figure 6:**
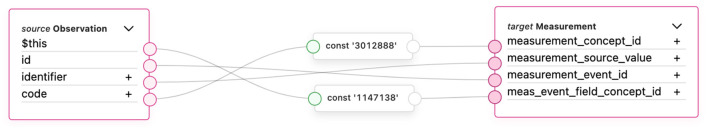
Creation of identifiers during blood pressure transformation in TermX.

Figure 6 illustrates how the identifier and inter-record relationship between the blood pressure measurements are implemented in TermX.

All other columns are transformed according to the same logic as in the general Measurement table transformation. Together, these transformations demonstrated that both simple and composite vital sign observations can be systematically converted into OMOP-compliant records using TermX, supporting reusable, rule-based transformation patterns for diverse clinical data types.

### 5.5 Vital signs transformation from OMOP CDM to FHIR

This subsection discusses the reverse transformation direction from OMOP CDM to FHIR, focusing on the transformation of vital signs. Body temperature and blood pressure are presented as examples for this process.

The transformation from OMOP CDM to FHIR supports use cases such as transformation validation, synthetic test data generation, and data exchange in FHIR-based environments. The clinical usability and broader implications of this transformation direction are discussed in Chapter 7.2.

#### 5.5.1 Transforming body temperature data

Body temperature follows the same transformation pattern as other single-value vital signs, including respiratory rate, heart rate, oxygen saturation, weight, height, head circumference, and body mass index.

**Figure 7:**
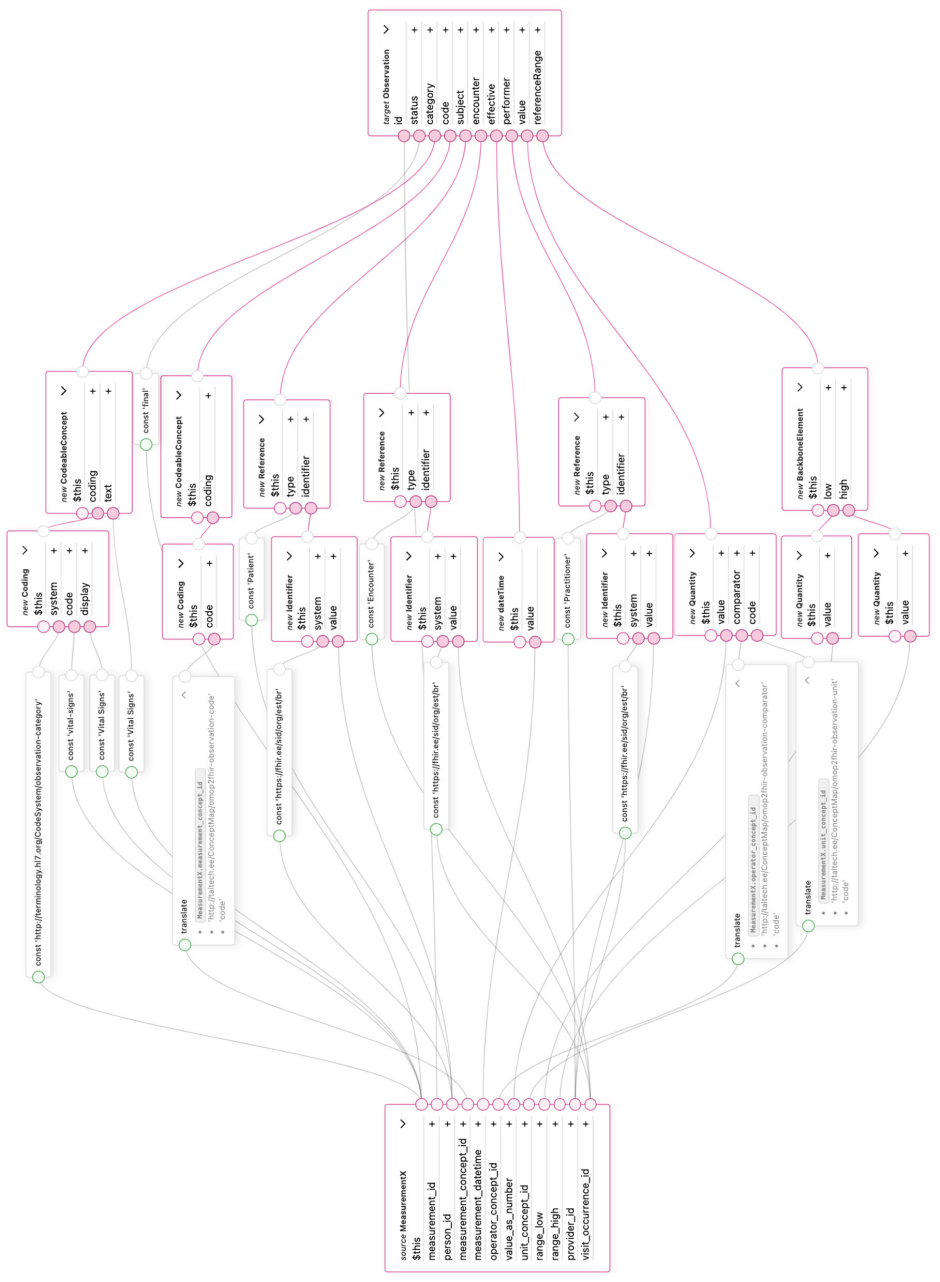
Transformation of OMOP CDM Measurement table to FHIR Observation resource in TermX.

A dedicated transformation component is created to convert OMOP CDM *Measurement* records into a FHIR *Observation* resource (Figure 7). The core fields, such as measurement value and date, are mapped directly because their data types and semantic meanings align between the two standards.

Additional clarifications regarding the mapping logic in Figure 7 are as follows:

In the transformation, several values are converted directly, as their data types and semantic meanings correspond to the elements being transformed. One such example is the primary key, which has an integer data type in OMOP CDM and can be transformed into the string identifier used in a FHIR resource.

In addition, the mandatory *status* element of the FHIR Observation resource is populated with the constant value “final.” This assumption is justified because OMOP CDM only stores completed measurement results. Data describing the category of the resource as a vital sign has also been added.

For transforming resource references, the *system* and *type* values are added as constants to ensure compatibility with the FHIR structure. However, these are not functional resource references in practice because OMOP CDM uses anonymized keys. During reverse transformation, due to de-identification requirements, the original references are not reconnected via relationship tables.

For terminology values, the Translate function is used together with a ConceptMap to avoid transforming the term exactly as it appears in the source data. This is necessary because, when transforming back from OMOP CDM to FHIR, the original terminology system of the data is unknown, and therefore, it is not guaranteed that the term values are compatible with HL7 terminology.

#### 5.5.2 Transforming diastolic and systolic blood pressure data

Blood pressure presents a more complex challenge because a single FHIR Observation contains two values (systolic and diastolic), but OMOP CDM stores them as two separate Measurement records. Therefore, the reverse transformation must merge these two records into a single Observation.

The blood pressure transformation is divided into two separate stages. In the first stage, the systolic and diastolic measurement results are transformed into two intermediate FHIR Observation resources. This is a temporary result in the transformation process and uses the same transformation component described in the previous subsection. These two intermediate FHIR resources are then grouped into a FHIR Bundle, which makes it possible to combine multiple FHIR resources into a single logical unit (44). By using a Bundle, it becomes possible to overcome the limitation of FML and transform two input records into one consolidated FHIR Observation resource. Before the first stage, the records are linked using the measurement_id, measurement_concept_id, and measurement_event_id fields to correctly match the corresponding systolic and diastolic values.

**Figure 8:**
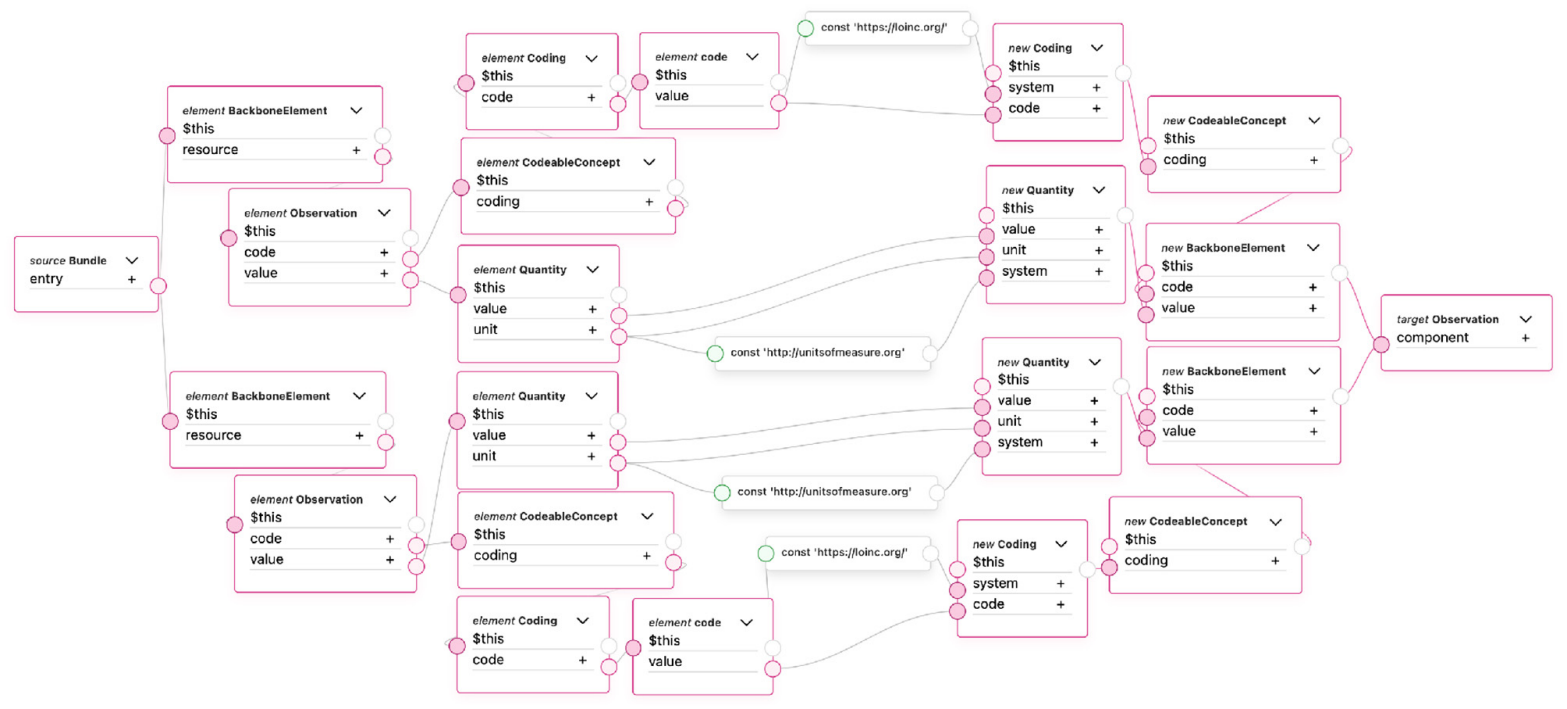
Blood pressure transformation from OMOP CDM to FHIR Observation resource in TermX.

Figure 8 illustrates the second stage of the transformation, where the two FHIR Observation resources connected via the Bundle are converted into a single FHIR Observation resource. The transformation uses data from both source resources to construct the final Observation. Since FHIR represents blood pressure values in the *Observation.component* structure, the values obtained from the two OMOP CDM Measurement records (systolic and diastolic blood pressure) are placed into the *Observation.component.value* elements of the combined resource. The figure specifically shows the transformation of this structure.

For the remaining resource elements, where the values in both source resources are identical, one of the source resources is used, and the matching fields are mapped directly to the corresponding elements in the final resource.

## 6 Evaluation and validation of the transformation process

This section evaluates the correctness and limitations of the implemented transformations based on the validation approach described in Section 4.1.3. The evaluation focuses on structural conformity and semantic accuracy, with particular emphasis on representative vital sign transformations.

In Design Science terms, this evaluation provides evidence for criterion characteristic validity by assessing whether the characteristics of the transformation outputs conform to the structural and semantic requirements of the target data model.

The evaluation covers both general FHIR-to-OMOP CDM table mappings and two representative vital sign transformations—body temperature and blood pressure— which illustrate single-value and composite measurement transformations. The results highlight areas of structural conformity, semantic preservation, and observed limitations.

### 6.1 Evaluation criteria

In this study, transformation accuracy is defined as conformance to the structural and semantic requirements of the target data model. Accuracy is assessed using quantitative indicators where applicable and qualitative analysis where quantitative measurement is not meaningful.

For the HL7 FHIR–to–OMOP CDM transformation, accuracy is measured quantitatively using structural completeness metrics, including the number of transformed columns, the number of untransformed mandatory columns, and the resulting mapping percentage. This approach is feasible because the OMOP CDM is a fixed relational schema with explicitly defined mandatory fields, allowing objective measurement of structural coverage.

In contrast, the OMOP CDM–to–FHIR transformation targets a flexible, resource-based data model. Due to the hierarchical structure of FHIR resources and the absence of a fixed set of mandatory elements comparable to relational schemas, column-level completeness metrics are not applicable in this direction. Consequently, accuracy for OMOP-to-FHIR transformations is evaluated qualitatively through semantic preservation and structural feasibility of the resulting FHIR resources.

This paper does not aim to statistically estimate transformation accuracy across large datasets but to demonstrate correctness and feasibility of the transformation logic at the level of formally defined model requirements.

Conformance was assessed by systematically comparing the transformed outputs against the formally defined structural and semantic requirements of the target data models. For OMOP CDM outputs, this included verification of mandatory fields, data types, foreign key relationships, and measurement semantics as defined in the OMOP CDM specification. For FHIR outputs, validation focused on the presence and correctness of mandatory resource elements, structural compliance with the Observation resource definition, and appropriate representation of composite measurements. Deviations from full conformance were explicitly documented and justified as prototype-level limitations. Data loss is evaluated as the absence of information that cannot be reconstructed in the target model. This includes missing terminology metadata and patient-level context that is not retained in the OMOP CDM representation.

Together, these criteria provide a structured basis for evaluating transformation correctness and usability at the proof-of-concept stage.

### 6.2 Structural conformity

Structural conformity was evaluated by verifying whether the transformed data satisfy the target data model requirements, including data types, mandatory fields, and structural relationships between tables.

For single-value measurements such as body temperature, validation confirmed that OMOP CDM Measurement fields are populated according to the target data types. Fields such as measurement_date, measurement_datetime, and measurement_time originate from a FHIR dateTime type and can be converted during later ETL stages. The value_as_number, range_low, and range_high fields are correctly transformed from FHIR valueQuantity and referenceRange elements. Foreign key fields (person_id, provider_id, and visit_occurrence_id) are populated correctly, ensuring proper linkage across tables. The mandatory measurement_concept_id and measurement_id fields are not instantiated at runtime in the prototype implementation but are assumed to be resolvable via terminology services and surrogate key generation in a production ETL pipeline.

For composite measurements such as blood pressure, validation confirmed that a single FHIR Observation is correctly transformed into two OMOP CDM Measurement records representing systolic and diastolic pressure. These records are structurally complete and correctly separated. Shared linkage fields, including measurement_source_value and measurement_event_id, preserve the association between the two measurements, while other fields follow the same transformation logic as in single-value measurements.

In the reverse transformation direction (OMOP CDM to FHIR), most OMOP CDM table fields can be mapped successfully to a FHIR Observation resource. To satisfy FHIR validity requirements, mandatory elements such as status and category are populated with fixed default values (e.g., final and vital-signs). Resource identifiers and references follow standard FHIR conventions. However, certain terminology-related elements, such as system URIs and human-readable labels in Observation.code, cannot always be reconstructed due to information loss inherent in the OMOP CDM representation.

### 6.3 Semantic accuracy

Semantic accuracy was evaluated by assessing whether the clinical meaning of the source data is preserved in the transformed representation.

For body temperature transformations, the original FHIR Observation meaning is retained. Temperature values are correctly transferred to value_as_number and value_source_value fields, and units are accurately preserved in unit_source_value. Foreign key references maintain contextual relationships between patient, provider, and encounter. Although concept identifier fields are not populated at this stage, the resulting records remain semantically valid representations of temperature measurements.

For blood pressure transformations, systolic and diastolic measurements are correctly distinguished using their respective LOINC codes (8480-6 and 8462-4) and mapped to the appropriate OHDSI concept identifiers. Measured values and units are preserved without rounding or loss. In the OMOP CDM–to–FHIR transformation, both measurements are correctly combined into the Observation.component structure of a single FHIR Observation resource. The associated terminology codes retain their intended meaning, and the resulting Observation represents a clinically correct blood pressure measurement. However, the human-readable text value cannot be reliably populated due to ambiguity between the two component measurements.

### 6.4 Summary of evaluation results

The validation results demonstrate that TermX enables structurally and semantically consistent bidirectional transformations between FHIR and OMOP CDM for key vital sign use cases. From FHIR to OMOP CDM transformations, minor issues related to the terminology translation function are identified, but these do not compromise the integrity of the core mappings. The prototype achieved Technology Readiness Level 3 (TRL 3) (39), corresponding to a proof-of-concept system operating reliably in a controlled environment.

In the reverse transformation direction (OMOP CDM to FHIR), challenges are identified that stem from structural differences between the standards: FHIR resources contain more patient-level information than is retained in OMOP CDM. Thus, although the transformations are technically feasible, certain data elements could not be reconstructed, leading to inherent information loss.

These findings substantiate the study's criterion claim by demonstrating that the proposed transformation components produce structurally and semantically valid representations of vital sign data, consistent with proof-of-concept validation at TRL 3.

Overall, the evaluation confirmed the feasibility of using TermX for rule-based FHIR-to-OMOP and OMOP-to-FHIR transformations and established a foundation for scaling the approach to broader clinical datasets and terminology systems.

## 7 Analysis and discussions

This chapter presents an analysis of the transformation results and the practical applicability of the developed solution. Section 1 evaluates the implemented transformation process and responds to Research Question 1. The sections that follow investigate the key technical and semantic challenges observed, providing insight into Research Question 2.

### 7.1 Transformation process from FHIR to OMOP CDM

This section addresses the first research question and divides the bidirectional transformation between HL7 FHIR and OMOP CDM into two parts. The first part focuses on the FHIR to OMOP CDM transformation, and addresses the research question: What are the accuracy, data loss, and usability characteristics of the transformations between HL7 FHIR and OMOP CDM?

The results show that transformations from HL7 FHIR to OMOP CDM are feasible, and that the resulting OMOP CDM table records are populated reliably. However, some data loss occurred during the transformation process. This is mainly caused by the different purposes and structures of the two standards. Therefore, the observed data loss is not the result of the transformation process itself, but of the conceptual and structural differences between the source and target standards.

The results of this paper differ from the guidance provided within the Vulcan project in several respects. The Vulcan documentation identifies correspondences between elements of FHIR resources and various OMOP CDM tables, but it does not define concrete transformation logic for any specific OMOP CDM table. As a result, the mappings presented by the project remain at a descriptive level and do not constitute a complete or directly applicable set of transformation rules.

Unlike the Vulcan project, which bases its transformation work on the *US Core Data for Interoperability* FHIR resource structure (21), the transformations developed in this study rely on the FHIR base resource structure. Basing the transformations on the base resource enables greater flexibility and reusability, as it is internationally applicable and does not depend on locally customized implementations. This approach makes it possible to use the developed solution as a general foundation for transforming data from FHIR to OMOP CDM and to adapt it as needed to meet the requirements of a specific organization or project. In addition, the reusability of the solution allows the same transformation logic to be applied at different stages of a project, for example, when incrementally extending an OMOP CDM–based database with new data. This also simplifies the task of keeping an existing OMOP CDM database up to date.

To assess the accuracy and completeness of the transformations, a column-level analysis is performed. Values obtained from FHIR resources are compared with the corresponding OMOP CDM table fields in terms of data type and semantic meaning. The results are presented in Table 3, which shows the number of successfully transformed columns and the number of mandatory columns that are not transformed.

**Table 3:** Completeness of OMOP CDM tables transformed from FHIR resources.

**OMOP CDM table**	**Transformed columns/total columns**	**Untransformed mandatory columns**	**Mapping percentage**
Care Site	5/6	0	83%
Provider	7/13	0	54%
Person	11/18	2	61%
Visit Occurrence	15/17	1	88%
Measurement	18/22	1	82%

The results in Table 3 show that most mandatory and optional columns in the OMOP CDM tables are successfully populated during the transformations, meaning that their correctness is validated. The untransformed columns are mainly due to missing information in the FHIR resources. The challenges related to the mapping of mandatory columns are discussed in more detail in the next subsections.

#### 7.1.1 Answer to research question 1 (FHIR to OMOP CDM)

The results indicate that transformations from HL7 FHIR to OMOP CDM are largely accurate and usable, with most mandatory and optional OMOP CDM fields populated correctly. Certain data elements could not be represented in OMOP CDM due to conceptual and structural differences between HL7 FHIR and OMOP CDM, resulting in partial information loss that is inherent to these differences rather than the transformation process.

### 7.2 Transformation process from OMOP CDM to FHIR

This section continues to address the first research question. The second part focuses on the OMOP CDM to FHIR transformation and evaluates the accuracy, data loss, and usability characteristics of the resulting transformations.

The transformation from OMOP CDM to FHIR is not symmetrical with the transformation from FHIR to OMOP CDM. This is due to the structural and granularity differences between the two standards. Therefore, only parts of the original FHIR resource can be restored during reverse transformation, and the fully original FHIR resource, suitable for use in clinical workflows, cannot be fully reconstructed.

The main reason is that metadata and some relationships present in the original FHIR resource are lost during the transformation to OMOP CDM. In addition, personally identifiable information associated with the FHIR resource cannot be restored. Due to OMOP CDM's de-identification principles, mandatory elements for a usable FHIR Patient resource, such as patient name, national identifier, or contact information, are not present in OMOP CDM. These elements are critical for the clinical usability of FHIR resources.

Despite these limitations, the reverse transformation has several practical use cases:

- Transformation validation: converting OMOP CDM back to FHIR enables verification of which values are lost, changed, or preserved during the process. This helps assess transformation quality and consistency.
- Synthetic test data generation: FHIR resources can be generated from OMOP CDM for use in development, validation, and performance testing. Because the transformed data remain de-identified and structurally realistic, they are suitable in situations where the use of real patient data is not ethically or legally permitted.
- Data exchange in FHIR-based environments: transformed OMOP CDM data can be used in HL7 FHIR based systems for interoperability purposes. For example, the MENDS-on-FHIR pilot project in the United States demonstrated the conversion of OMOP CDM data into FHIR to support data exchange for chronic disease surveillance in FHIR-based infrastructure (45).

If a legally and ethically compliant mechanism is available to securely link OMOP CDM data with personal identifiers during reverse transformation, this could improve transparency in research data usage. For example, individuals could be informed about whether and how their data have been used in scientific studies. Such an approach would strengthen trust in research and could encourage people to allow their data to be used in future health studies. This solution faces ethical implications—particularly concerning data de-identification and the risk of re-identification during reverse transformation—and requires further research to evaluate risks and limitations.

#### 7.2.1 Answer to research question 1 (OMOP CDM to FHIR)

The results show that transformations from OMOP CDM to HL7 FHIR are inherently limited in accuracy and clinical usability due to structural, granularity, and de-identification differences between the two standards. While some elements can be reconstructed for validation, testing, and interoperability purposes, metadata, relationships, and personally identifiable information cannot be restored. Consequently, these transformations are suitable for secondary use cases but do not support full reconstruction of clinically usable FHIR resources.

### 7.3 Challenges in transformations between FHIR and OMOP CDM

This section addresses the second research question: What are the key technical and semantic interoperability challenges in bidirectional transformations between FHIR and OMOP CDM? The analysis is structured into multiple subsections, each focusing on a distinct challenge.

#### 7.3.1 Challenge 1: creating primary keys for OMOP CDM tables

One of the main technical challenges in transforming data from FHIR to OMOP CDM is the conversion of OMOP CDM's integer-based primary keys.

FHIR resources use three different types of identifiers (46): “Business Identifier,” “Canonical URL,” or “Location URL.” These identifiers are of the *string* data type. FHIR identifiers, in their original form, cannot be used directly as primary keys in OMOP CDM tables because this would conflict with OMOP CDM's de-identification principles, as it could allow the re-identification of individuals.

In addition, converting string-based identifiers into integers during transformation does not guarantee uniqueness or consistency, as different string values may map to the same numeric value. This can lead to non-unique keys, table conflicts, and compromised referential integrity, while also eliminating the original identifier and hindering validation and traceability. Although numeric keys could be generated from text identifiers using a dedicated algorithm, the current solution lacks FML support for such functionality.

Therefore, two alternative solutions are considered in this paper. The first option is to use the FHIR *Extension* mechanism, which allows additional structured data to be attached to a resource that is not part of its base definition. This mechanism could be used to add a stable integer identifier to each resource, compatible with both OMOP CDM's primary key data type and its de-identification requirements. This would enable a stable link between a FHIR resource and its corresponding OMOP CDM table record. Using such extensions requires the data sender to manage the association between this integer identifier and the person or related primary key in their own data.

The second approach, which is used in this paper, is to store the business identifier of the FHIR resource in the *_source_value column of the OMOP CDM tables. Based on these stored identifiers, a relationship table can later be created during the next ETL phase to generate stable integer primary keys. The business identifier is chosen because it remains constant over time and is suitable for use outside the FHIR environment.

The advantage of this approach is that the creation of the corresponding relationship tables remains the responsibility of the OMOP CDM database administrator. This ensures better data compatibility when data are received from multiple sources. It also helps to avoid duplicate data, as the generation of primary keys can include a check to verify whether a record with the same identifier already exists.

Members of the Vulcan project have noted that using the _source_value column for transformations may limit its potential for supporting data traceability (47). However, the OMOP CDM documentation specifies that this column should not be used in standard analytical processes, as its values are not consistent across data sources (15). Therefore, this paper followed the OMOP CDM documentation: the _source_value column is used only for establishing relationships, but is not populated with values in later transformation stages.

#### 7.3.2 Challenge 2: creating the OMOP CDM reference system

The second technical challenge is the creation of a reference system between OMOP CDM tables. In FHIR, resource references are expressed in string-based format, whereas in the physical data model of OMOP CDM, references are represented as integer foreign keys.

This paper followed the logical model from the Vulcan Implementation Guide for OMOP CDM (20), where foreign key columns are defined using the FHIR-compatible *Reference* data type. This approach makes it possible to transform a FHIR resource reference into a foreign key in the OMOP CDM table. Similarly to the creation of primary keys, a relationship table can later be created to store the corresponding integer-based foreign key in the OMOP CDM table. As with primary keys, the keys used in OMOP CDM must maintain the de-identification of individuals.

The authors of this article (73) and the Vulcan project have also recommended using two parallel databases: the first containing the OMOP CDM-based database, and the second storing the relationships between FHIR identifiers and OMOP CDM keys (21). This separation ensures the required level of de-identification and supports secure data management.

The method for linking a resource reference to its corresponding primary key depends on the type of resource reference used in the source data. One possible approach considered is to use FHIR Extensions, similar to the first method proposed for primary keys. However, this would mean that the sender of the FHIR data would also be responsible for managing and maintaining these keys and their associations. Such an approach would reduce the flexibility of an integrated OMOP CDM based database, particularly when combining data from multiple sources, since each data provider would maintain its own relationship tables.

The second approach, and the one used in this paper, is to create foreign keys based on logical resource references. The value used in a logical reference can be linked to a business identifier, making it a preferred solution. Linking foreign keys to business identifiers provides a complete reference system because the relationship tables remain under the control of the OMOP CDM database administrator, and because the business identifier itself is stable. According to the HL7 FHIR standard, data providers can include logical references in their FHIR data, which does not impose any additional administrative burden, such as creating or maintaining separate relationship tables.

The resulting reference system is therefore technically feasible and well-suited for use in the transformation process.

#### 7.3.3 Challenge 3: untransformed mandatory OMOP CDM columns

During the transformation process, one of the challenges is populating mandatory fields in the OMOP CDM data model in cases where the corresponding information is missing in the HL7 FHIR base resources. In total, four mandatory columns remained untransformed.

Two of these columns belong to the *Person* table and are related to a person's race and ethnicity. These data elements are not included in the HL7 FHIR base resource, but these could be added using *Extension* fields. The other two untransformed columns are measurement_type_concept_id and visit_type_concept_id, which in OMOP CDM define the type of record origin (type concept) (48). FHIR resources do not directly store this information; however, considering the specific transformation context, it is possible to infer values for these fields. Therefore, while these fields cannot be automatically populated in a fully reusable solution, they can be added in context-specific transformations.

In addition, several non-mandatory OMOP CDM columns also remained unfilled because their values are not available in the source data nor necessary from an analytical perspective. These are not discussed in detail here, as they do not affect the overall quality or applicability of the transformations.

#### 7.3.4 Challenge 4: limitations of terminology mappings

Mapping terminologies between HL7 FHIR and OMOP CDM is not a purely mechanical task—it requires semantic analysis of concept meanings and careful alignment of equivalent terms. Although the OHDSI vocabulary includes several widely adopted medical terminologies also used in FHIR, the two are not fully compatible in all cases. Previous research has highlighted the complexity of terminology alignment, and this challenge remains only partially resolved in this paper. This paper does not aim to provide a comprehensive solution for terminology harmonization between HL7 FHIR and OMOP CDM; instead, it focuses on identifying practical constraints and trade-offs encountered when implementing partial, bidirectional terminology mappings in a tool-based transformation.

In this work, one of the main difficulties is selecting an appropriate ValueSet for HL7 FHIR data (49). Although FHIR resources often reference specific code systems, a precise ValueSet is not always defined, which complicates the creation of reusable transformations. Achieving full terminological coverage would therefore require extensive effort, as term mappings would have to be established for multiple potential ValueSets. For this reason, this paper primarily focused on mapping standard terminology terms, and in several cases, only partial mappings are created. This is done to demonstrate the technical feasibility of using concept mapping tables in the transformation process.

Another complicating factor in bidirectional mappings is that when data are transformed not only from FHIR but also from other sources (e.g., CDA), the term being transformed may not be compatible with HL7 terminology at all. Therefore, this paper concluded that bidirectional terminology mappings should be implemented through explicit concept mapping tables rather than using the *_source_value fields. This approach provides greater control and transparency, ensuring better reusability and validation of the solution. However, it also requires extensive manual work—reviewing, cataloging, and partially mapping OHDSI terminology terms to those that can be aligned with HL7 terminologies.

Furthermore, reverse transformations must take into account that FHIR *CodeableConcept* fields contain not only the term code but also the system (terminology URI) and text (human-readable label) values. Although the term code itself can be restored from OMOP CDM using a concept mapping table, the system and text values cannot be recovered in this way. These could only be re-derived if the transformation context and the original terminology source are both known, which cannot be guaranteed in a fully reusable transformation solution.

#### 7.3.5 Challenge 5: combining multiple structures in a one-way transformation

During the transformation process, a challenge arose in situations where one input structure had to be transformed into two target structures, or conversely, two input structures had to be combined into a single target structure. This issue appeared most prominently in the transformation of blood pressure data. In HL7 FHIR, the *Observation* resource contains both systolic and diastolic values within a single resource, whereas in OMOP CDM, these values are stored as two separate records in the *Measurement* table.

When addressing this complexity, certain limitations are identified in the FHIR Mapping Language (FML) functionality of the tool. FML allows only one input and one output structure to be defined per transformation (41). One possible solution considered is to use the FHIR Bundle resource, which serves as a container for grouping multiple FHIR resources and transmitting them as a single unit (44). However, using a Bundle is not suitable in this case, since it can only group FHIR resources, while the transformation from FHIR to OMOP CDM requires creating two OMOP CDM table records.

To overcome this limitation, a two-step solution is being developed: the single FHIR resource is transformed into two OMOP CDM records using two separate transformation components. These transformation components are distinguished by the terminology code used in the FHIR base resource, which allows systolic and diastolic blood pressure results to be identified separately. This distinction is necessary because FML does not support conventional conditional statements such as *if*, *when*, or *else*. Instead, each conditional logic must be implemented as an independent transformation rule based on a corresponding filter.

A similar challenge also occurred during the reverse transformation. Although the logic differed, it is solved using a two-step approach. In the reverse direction (from OMOP CDM to FHIR), it is technically possible to use the FHIR Bundle. For this, the OMOP CDM Measurement table records must first be converted into intermediate FHIR resources containing the systolic and diastolic blood pressure results separately. These intermediate resources can then be combined into a single blood pressure Observation within a FHIR Bundle. To ensure that the correct pairs of systolic and diastolic measurements are matched, the Measurement table records must first be linked through the meas_event_id column.

Although two-step solutions add complexity and workload to the overall ETL process, the current limitations of the FML tool make it impossible to simplify this process further. In practical ETL process implementation, it is particularly important to ensure that the reverse transformation produces correctly matched systolic and diastolic blood pressure pairs.

#### 7.3.6 Answer to research question 2

The analysis identified several key technical and semantic interoperability challenges in transformations between HL7 FHIR and OMOP CDM, including primary and foreign key generation, reference management, incomplete population of mandatory fields, terminology alignment, and structural mismatches between the standards. Many of these challenges arise from fundamental differences in standards, de-identification requirements, and semantic granularity rather than from tooling limitations alone. While implementation approaches exist, most require context-specific decisions, additional metadata management, or multi-step transformation processes.

## 8 Related work

Several international initiatives and working groups have addressed the development of transformation processes between OMOP CDM and HL7 FHIR, the most prominent of which are OHDSI and the Vulcan HL7 project (21). These initiatives have laid the foundation for standardization efforts and interoperability between health data models. This section also highlights the challenges encountered in transformation processes described by other authors.

### 8.1 International initiatives and working groups

The OMOP CDM and its standardized terminology are governed by OHDSI, which has established a broad international network to support the implementation, validation, and federated analysis of the data model (19). The tools developed by OHDSI (50) support database structure creation, terminology management (e.g., Athena), data quality assessment (e.g., DataQualityDashboard), and the execution of standard analyses (e.g., Atlas). OHDSI itself does not provide direct support for transforming data from the HL7 FHIR standard or from other healthcare data standards into the OMOP CDM.

One of the most significant international initiatives in the field of interoperability between HL7 FHIR and OMOP CDM is the Vulcan HL7 Project (*Vulcan HL7 Project*, hereinafter the Vulcan project), which operates through collaboration between the HL7 organization and the OHDSI community (21). The broader objective of the Vulcan project is to support the transformation of FHIR data into OMOP CDM. The transformation documentation developed within the Vulcan project is based on the *US Core Data for Interoperability* (51) FHIR resource structure, which is treated within the project as the primary source format, along with the corresponding terminology *SNOMED CT International Patient Summary* (52).

Within the Vulcan project, the first official implementation guide to support transformation from FHIR to OMOP CDM is currently being published in draft status (20). Its purpose is to provide reliable transformation rules, thereby reducing the cost and time required for the transformation process. To date, no ready-made tool or workflow has been developed that enables data transformation from the HL7 FHIR standard to OMOP CDM or vice versa.

Both of these international networks—OHDSI and the Vulcan project—actively contribute to improving interoperability between standards, and regular meetings and discussions take place within their respective working groups. The HL7 FHIR and OMOP CDM standards are both widely used internationally; however, their mutual interaction and interoperability have so far received limited attention in the scientific literature (53).

### 8.2 Existing challenges in mapping

The integration of FHIR with the OMOP CDM faces several challenges. These challenges arise from fundamental differences in the design goals of the standards.

A key challenge lies in the different ways each model represents and structures data. FHIR relies on a resource-based model that emphasizes the exchange of discrete data elements. OMOP CDM, however, uses a more normalized relational database structure suited for comprehensive, longitudinal data analysis (54, 55). These differences require extensive mapping and transformation to ensure accurate representation in both formats. Converting clinical data from FHIR to OMOP CDM often involves a complex ETL process that must handle variations in data granularity and coding systems (56). If these ETL process steps are not executed precisely, they can lead to data loss or misinterpretation.

Despite the popularity of both standards, only a limited number of academic studies have deeply documented the ETL processes for FHIR-to-OMOP conversions (22). Because FHIR is a platform specification that requires local adaptation, implementations often diverge; without shared Implementation Guides, mappings are hard to generalize or reuse (57).

Some of the main challenges include (20, 34, 58):

- Data type incompatibilities: OMOP CDM uses numeric identifiers, whereas FHIR uses strings and URLs for referencing resources.
- Structural mismatches: not all FHIR resources have one-to-one equivalents in OMOP CDM, and vice versa.
- Incomplete data: mandatory fields in OMOP CDM might not always have equivalents in FHIR instances, and vice versa.
- Terminology alignment: although both support standards like SNOMED CT and LOINC, differences in granularity and national extensions can hinder direct mappings (59).

The quality of source data also affects data transformation quality. EHRs can differ significantly across institutions, which makes it challenging to ensure reliable data during the transformation process (56, 60). In addition, privacy regulations require careful data handling, which can complicate data sharing among different healthcare entities (61).

Interoperability between FHIR and OMOP CDM is further hindered by inconsistent vocabularies and terminologies. FHIR supports coding systems like SNOMED and LOINC, while OMOP CDM has its own standardized vocabularies (55, 62). Mapping these terminologies demands significant effort and often requires specialized expertise to maintain accuracy (63). Integrating FHIR with OMOP CDM involves multiple challenges related to data structure, data quality, terminology alignment, and infrastructure. Overcoming these obstacles requires healthcare stakeholders to collaborate on standardized protocols and tools that support efficient data exchange and interoperability between these two essential models.

### 8.3 Research gaps and the need for tool-based solutions

One major gap is the lack of clear, comprehensive methods for the transformation process. Although various studies have proposed frameworks and tools for converting data from OMOP CDM to FHIR, such as the Clinical Asset Mapping Program for FHIR (CAMP FHIR) (64), there is no standardized, detailed guidance on how to execute these transformations effectively. Henke et al. (65, 66) note that existing literature does not offer a consistent process for harmonizing source data into the OMOP CDM. As a result, data quality and interoperability can vary, which ultimately affects research outcomes.

Another challenge is the complexity of ETL processes. As Unberath et al. (67) point out, integrating additional data items into OMOP CDM can be difficult, and incremental loading of data remains an open issue. Reinecke et al. (60) have also emphasized the need for more rigorous quality assurance. Furthermore, Henke et al. (65) highlight the absence of a generic approach to handling data changes over time, suggesting that more robust frameworks are needed for dynamic data environments.

Interoperability between different data models and standards is another critical gap. Although FHIR is seen as a meta-model for unifying various common data models (including OMOP) (68), real-world implementations often lack clarity and efficiency. Essaid et al. (45) underscore the importance of effective batch conversion methods, yet current tools may not fully address the complexities of practical applications. In addition, Alkarkoukly et al. (69) show that these transformations often rely on open-source tools, highlighting a need for solutions that do not demand extensive technical expertise.

While important strides have been made in FHIR-to-OMOP transformations, notable gaps remain in methodological consistency, ETL-process complexity, interoperability, and handling specialized data. Addressing these shortcomings is essential for improving data transformation processes and, ultimately, healthcare research and outcomes.

## 9 Conclusion and future work

The secondary use of health data, including its application in research, requires standardized and machine-readable data exchange. This paper addresses the transformation of vital signs data between the HL7 FHIR standard and the OMOP Common Data Model (CDM). The developed reusable transformation components simplify the future implementation of similar mappings and support the development of full ETL process to support the secondary healthcare data use.

The objective of the paper is to design a prototype enabling the transformation of vital signs in both directions between HL7 FHIR and OMOP CDM. A set of bidirectional and reusable transformation rules are developed using the FHIR Mapping Language (FML) and TermX visual editor, and the technical and semantic challenges associated with such transformations are identified and analyzed. Initial manual testing and validation are carried out to confirm the feasibility of the approach.

The research followed the Design Science methodology, which enabled an iterative development process, from problem investigation to the creation of a functional software artifact, and the assessment of its practical value. The resulting solution achieved Technology Readiness Level 3 (TRL 3), indicating proof-of-concept validation in a controlled environment.

The evaluation showed that transformations between HL7 FHIR and OMOP CDM introduce several challenges, including the creation of primary and foreign key systems based on FHIR identifiers and references, information loss due to differences in model granularity, terminology alignment, and structural limitations imposed by available tooling. Despite these challenges, this paper demonstrated that technically feasible and semantically meaningful transformations are achievable. Further development is required to achieve a fully operational implementation of the prototype.

A first direction for future work is the application of the transformation components to real clinical datasets. This requires the construction of a complete ETL process, including alignment with the physical OMOP CDM data model and integration with clinical data sources.

A second key development need is systematic and large-scale validation of component functionality within real-world operational workflows. Such validation would make it possible to assess the suitability of the solution for production environments and advance the technology readiness level of the approach.

A third direction is the expansion of transformation rules to cover additional HL7 FHIR resources and OMOP CDM table structures. The goal is to support key clinical domains such as medications, laboratory results, and diagnoses. This would enable the creation of a broader transformation framework capable of supporting bidirectional conversion between HL7 FHIR and OMOP CDM across diverse healthcare datasets.

In the longer term, these development paths will support the establishment of a high-quality OMOP CDM based database that enables the secondary use of health data in national and international research. Standardized data will foster data-driven decision-making, support evidence-based policy development, and promote innovation within the healthcare sector.

## Data Availability

The datasets presented in this article are not readily available because they are not applicable. The study does not generate or analyze shareable patient-level datasets. Requests to access the datasets should be directed to hanna.ardel@taltech.ee.

## References

[1] Thomason J. Data, digital worlds, and the avatarization of health care. Glob Health J. (2024) 8:1–3. doi: 10.1016/j.glohj.2024.02.003

[2] Forum WE. How to Harness the Power of Health Data to Improve Patient Outcomes. (2024). Available online at: https://www.weforum.org/stories/2024/01/how-to-harness-health-data-to-improve-patient-outcomes-wef24/ (Accessed March 01, 2025).

[3] World Economic Forum. 4 Ways Data is Improving Healthcare. (2019). Available online at: https://www.weforum.org/stories/2019/12/four-ways-data-is-improving-healthcare/ (Accessed March 01, 2025).

[4] Lutenski E. Healthcare Data Challenges (2025). Available online at: https://www.decentriq.com/article/healthcare-data-challenges (Accessed October 27, 2025).

[5] Kilgus T Patecka A Schurig T Kari A Gubser R Gersch M . Creating value from the secondary use of health data: International examples, best practices, and opportunities to scale. Commun Assoc Inf Syst. (2024) 55:507–34. doi: 10.17705/1CAIS.05520

[6] Zurynski Y Smith CL Vedovi A Ellis LA Knaggs G Meulenbroeks I . Mapping the Learning Health System: A Scoping Review of Current Evidence. Sydney: Australian Institute of Health Innovation and the NHMRC Partnership Centre for Health System Sustainability (2020). Available online at: https://researchers.mq.edu.au/files/134364432/Publisher_version_open_access_pdf

[7] PWC. Transforming Healthcare Through Secondary Use of Health Data. Falls Church, VA: PriceWaterhouseCoopers (2009).

[8] Boyd M Zimeta M Tennison J Alassow M. Secondary Use of Health Data in Europe. London: Open Data Institute (2021). doi: 10.61557/LDCD2421

[9] European Commission. Ettepanek: EUROOPA PARLAMENDI JA NÖUKOGU MÄÄRUS ühtse Euroopa Terviseandmeruumi Kohta. (2022). Available online at: https://eur-lex.europa.eu/legal-content/ET/TXT/?uri=CELEX:52022PC0197 (Accessed October 27, 2025). In Estonian.

[10] BC Platforms. EHDS Overview. (2024). Available online at: https://ehds.bcplatforms.com-of-ehds/ (Accessed October 27, 2025). In Estonian.

[11] Klementi T Piho G Ross P. A reference architecture for personal health data spaces using decentralized content-addressable storage networks. Front Med. (2024) 11:1411013. doi: 10.3389/fmed.2024.1411013 39081693 PMC11286498

[12] Wise J Möller A Christie D Kalra D Brodsky E Georgieva E . The positive impacts of real-world data on the challenges facing the evolution of biopharma. Drug Discov Today. (2018) 23:788–801. doi: 10.1016/j.drudis.2018.01.034 29337204

[13] Grimberg F Asprion PM Schneider B Miho E Babrak L Habbabeh A. The real-world data challenges radar: a review on the challenges and risks regarding the use of real-world data. Digit Biomark. (2021) 5:148–57. doi: 10.1159/000516178 34414352 PMC8339486

[14] Sydes MR Murray ML Ahmed S Apostolidou S Bliss JM Bloomfield C . Getting our ducks in a row: The need for data utility comparisons of healthcare systems data for clinical trials. Contemp Clin Trials. (2024) 141:107514. doi: 10.1016/j.cct.2024.107514 38537901

[15] ODHSI. The Book of ODHSI. (2021). Available online at: https://ohdsi.github.io/TheBookOfOhdsi/CommonDataModel.html (Accessed January 02, 2025).

[16] HL7. Resource Types. (2025). Available online at: https://hl7.org/fhir/resourcelist.html (Accessed February 28, 2025).

[17] Vlieghere C. How FHIR and OMOP are Competing Toward Healthcare Data Interoperability. (2024). Available online at: https://wwwtirohealth/resources/how-fhir-and-omop-are-competing-toward-healthcare-data-interoperability (Accessed March 23, 2025).

[18] Simon F Schladetzky J Macke S Ablaß T Ingenerf J Kock-Schoppenhauer AK. Metadata Driven Integration of Clinical Data for Secondary Use in FHIR - A Pilot Study at the UKSH. Amsterdam: IOS Press (2024). doi: 10.3233/SHTI240850 39234717

[19] ODHSI. Standardized Data: The OMOP Common Data Model. (2025). Available online at: https://www.ohdsi.org/data-standardization/ (Accessed January 25, 2025).

[20] HL7 Vulcan. FHIR to OMOP FHIR IG. (2024). Available online at: https://build.fhir.org/ig/HL7/fhir-omop-ig/index.html (Accessed February 28, 2025).

[21] Project HV. 2025 Vulcan Project Proposal. (2025). Available online at: https://confluence.hl7.org/spaces/VA/pages/325451882/2025+Vulcan+project+proposal (Accessed April 02, 2025).

[22] Peng Y Henke E Reinecke I Zoch M Sedlmayr M Bathelt F. An ETL-process design for data harmonization to participate in international research with German real-world data based on FHIR and OMOP CDM. Int J Med Inf. (2023) 169:104925. doi: 10.1016/j.ijmedinf.2022.104925 36395615

[23] TermX. TermX Official. (2024). Available online at: https://www.termx.org (Accessed October 30, 2025).

[24] Bossenko I Piho G Ross P. TermX: A Game Changer in the Healthcare Interoperability. Amsterdam: IOS Press (2024). p. 88–9. doi: 10.3233/SHTI240352 39176681

[25] Bossenko I Piho G Ivanova M Ross P. TermX: The Semantic Interoperability, Knowledge Management and Sharing Platform. Amsterdam: Elsevier (2024). p. 27. doi: 10.1016/j.softx.2024.101839

[26] Bossenko I. A Domain-Specific Framework for Supporting Semantic Interoperability in Primary and Secondary Use of Health Data on the Example of the Estonian National Health Information System (Doctoral thesis) (2025). Available online at: https://digikogu.taltech.ee/et/Download/82dc296e-6e60-48dd-bb87-ac0dc7aa47fd

[27] Wieringa R. Design Science Methodology for Information Systems and Software Engineering. Cham: Springer (2014). doi: 10.1007/978-3-662-43839-8 (Accessed April 11, 2025).

[28] Bossenko I Randmaa R Piho G Ross R. Interoperability of health data using FHIR mapping language: transforming HL7 CDA to FHIR with reusable visual components. Front Digit Health. (2024) 6:1480600. doi: 10.3389/fdgth.2024.1480600 39749099 PMC11693713

[29] HL7. FHIR Mapping Language. (2023). Available online at: http://hl7.org/fhir/mapping-language.html (Accessed October 27, 2025).

[30] Republic of Estonia Ministry of Social Affairs. RAPORT D: HAIGLAVÕRGU ARENGUKAVA 2040 Patsiendikeskne integreeritud haiglavõrgu arengukava (REFORM/SC2019/140). (2019). Available online at: https://www.sm.ee/sites/default/files/documents/2022-06/Inimkeskne%20ja%20integreeritud%20haiglav%C3%B5rk%20-%20IV%20Raport%20-%20EST.pdf (Accessed March 01, 2025). In Estonian.

[31] HL7. Introduction to HL7 Standards. (2025). Available online at: https://www.hl7.org/implement/standards/product_brief.cfm?product_id=496 (Accessed March 29, 2025).

[32] Health E Centre WIS. HL7 standardid. (2024). Available online at: https://teabekeskus.tehik.ee/et/standardid/hl7 (Accessed March 01, 2025). In Estonian.

[33] Health E Centre WIS. Uue põlvkonna Tervise Infosüsteem Visioon Tervise Infosüsteemile. (2021). Available online at: https://www.tehik.ee/sites/default/files/2021-11/upTIS%20visioon%2011.06.2021.pdf (Accessed March 01, 2025). In Estonian.

[34] ODHSI. OMOP CDM v5.4. (2025). Available online at: https://ohdsi.github.io/CommonDataModel/cdm54.html#Current_Support_for_CDM_v54 (Accessed February 28, 2025).

[35] HL7. Observation Vital Signs Panel Profile. (2023). Available online at: https://www.hl7.org/fhir/observation-vitalsigns.html (Accessed April 02, 2025).

[36] Sapra A Malik A Bhandari B. Vital Signs Assessment. National Center for Biotechnology Information (2023). Available online at: https://www.ncbi.nlm.nih.gov/books/NBK553213/

[37] Brekke IJ Puntervoll LH Pedersen PB Kellett J Brabrand M. The value of vital sign trends in predicting and monitoring clinical deterioration: a systematic review. PLoS ONE. (2019) 14:e0210875. doi: 10.1371/journal.pone.0210875 (Accessed May 02, 2025). 30645637 PMC6333367

[38] Larsen KR Lukyanenko R Mueller RM Storey VC Parsons J VanderMeer D . Validity in design science. MIS Q. (2025) 49:1267–94. doi: 10.25300/MISQ/2024/18064 PMC13004602 41868972

[39] Manning C. Technology Readiness Levels. (2023). Available online at: https://www.nasa.gov/directorates/somd/space-communications-navigation-program/technology-readiness-levels/ (Accessed April 12, 2025).

[40] Bossenko I Õitspuu R. Patsiendi Üldandmete Teenus/Master Patient Index. (2024). Available online at: https://fhir.ee/ig/mpi/1.1.1/site/artifacts.html (Accessed March 29, 2025). In Estonian.

[41] HL7. FHIR Mapping Language. (2025). Available online at: https://build.fhir.org/mapping-language.html (Accessed April 30, 2025).

[42] HL7. FHIRPath. (2025). Available online at: https://build.fhir.org/fhirpath.html (Accessed April 30, 2025).

[43] ODHSI. Search Terms. (2025). Available online at: https://athena.ohdsi.org/search-terms/start (Accessed October 29, 2025).

[44] HL7. Resource Bundle – Content. (2025). Available online at: https://build.fhir.org/bundle.html (Accessed May 03, 2025).

[45] Essaid S Andre J Brooks IM Hohman KH Hull M Jackson SL . MENDS-on-FHIR: leveraging the OMOP common data model and FHIR standards for national chronic disease surveillance. JAMIA Open. (2024) doi: 10.1101/2023.08.09.23293900 38818114 PMC11137321

[46] HL7. Base Resource Definitions. (2025). Available online at: https://build.fhir.org/resource.html (Accessed February 28, 2025).

[47] Project V. 2025 Identifier Mapping. (2025). Available online at: https://confluence.hl7.org/spaces/VA/pages/325452073/2025+Identifier+Mapping (Accessed October 29, 2025).

[48] OHDSI. Vocab.-TYPE_CONCEPTVocab. TYPE_CONCEPT. (2024). Available online at: https://github.com/OHDSI/Vocabulary-v5.0/wiki/ (Accessed April 19, 2025).

[49] HL7. Resource ValueSet – Content. (2025). Available online at: https://hl7.org/fhir/valueset.html (Accessed December 20, 2025).

[50] ODHSI. Observational Health Data Sciences and Informatics. (2025). Available online at: https://github.com/OHDSI (Accessed October 29, 2025).

[51] Interoperability Standards Platform. United States Core Data for Interoperability (USCDI). (2025). Available online at: https://www.healthit.gov/isp/united-states-core-data-interoperability-uscdi (Accessed April 02, 2025).

[52] SNOMED International. The International Patient Summary Terminology. (2025). Available online at: https://www.snomed.org/international-patient-summary-terminology (Accessed April 02, 2025).

[53] Reinecke I Zoch M Reich C Sedlmayr M Bathelt F. The Usage of OHDSI OMOP – A Scoping Review. Amsterdam: IOS Press (2023). 10.3233/SHTI210546 34545824

[54] Lenert LA Ilatovskiy AV Agnew J Rudisill P Jacobs J Weatherston D . Automated production of research data marts from a canonical fast healthcare interoperability resource data repository: applications to COVID-19 research. J Am Med Inf Assoc. (2021) doi: 10.1101/2021.03.11.21253384 33993254 PMC8243354

[55] Peng Y Nassirian A Ahmadi N Sedlmayr M Bathelt F. Towards the Representation of Genomic Data in HL7 FHIR and OMOP CDM. Amsterdam: IOS Press (2021). 1–9. doi: 10.3233/SHTI210545 34545823

[56] Ong TC Kahn MG Kwan BM Yamashita T Brandt E Hosokawa P . Dynamic-ETL: a hybrid approach for health data extraction, transformation and loading. BMC Med Inform Decis Mak. (2017) 17:134. doi: 10.1186/s12911-017-0532-3 28903729 PMC5598056

[57] HL7. Profiling FHIR. (2025). Available online at: https://build.fhir.org/profiling.html (Accessed October 19, 2025).

[58] HL7. Resource References. (2025). Available online at: https://build.fhir.org/references.html (Accessed May 03, 2025).

[59] HL7. HL7 Terminology Home Page. (2025). Available online at: https://terminology.hl7.org/ (Accessed October 29, 2025).

[60] Reinecke I Zoch M Wilhelm M Sedlmayr M Bathelt F. Transfer of Clinical Drug Data to a Research Infrastructure on OMOP – A FAIR Concept. Amsterdam: IOS Press (2021). doi: 10.3233/SHTI210815 34795082

[61] Hoffmann K Nesterow I Peng Y Henke E Barnett D Klengel C . Streamlining intersectoral provision of real-world health data: a service platform for improved clinical research and patient care. Front Med. (2024) 11:1377209. doi: 10.3389/fmed.2024.1377209 38903818 PMC11188485

[62] Bennett AM Ulrich H Van Damme P Wiedekopf J Johnson AE MIMIC-IV. on FHIR: converting a decade of in-patient data into an exchangeable, interoperable format. J Am Med Inform Assoc. (2023) 30:718–25. doi: 10.1093/jamia/ocad002 36688534 PMC10018258

[63] Hur K Lee J Oh J Price W Kim Y Choi E. Unifying heterogeneous electronic health records systems via text-based code embedding. In: Conference on Health, Inference, and Learning. PMLR (2022). p. 183–203. doi: 10.2196/preprints.32523

[64] Pfaff ER Champion J Bradford RL Clark M Xu H Fecho K . Fast Healthcare Interoperability Resources (FHIR) as a meta model to integrate common data models: development of a tool and quantitative validation study. Canada: *JMIR Med Inf*. (2019) 7:e15199. doi: 10.2196/15199 31621639 PMC6913576

[65] Henke E Zoch M Peng Y Reinecke I Sedlmayr M Bathelt F. Conceptual design of a generic data harmonization process for OMOP CDM. Preprints. (2023). doi: 10.20944/preprints202311.0104.v1 PMC10895818 38408983

[66] Henke E Zoch M Peng Y Reinecke I Sedlmayr M Bathelt . Conceptual design of a generic data harmonization process for OMOP common data model. BMC Med Inf Decis Making. (2024). doi: 10.1186/s12911-024-02458-7 38408983 PMC10895818

[67] Unberath P Prokosch HU Gründner J Erpenbeck M Maier C Christoph J. EHR-*Independent Predictive Decision Support Architecture Based on OMOP*. Stuttgart; New York, NY: Georg Thieme Verlag KG Stuttgart (2020). doi: 10.1055/s-0040-1710393 32492716 PMC7269719

[68] Jathissa P Rohatsch L Sauermann S Hussein R. OMOP-on-FHIR: A FHIR Server Development to Facilitate Data Interaction with the OMOP-CDM and FHIR for PGHD. In: Digital Health and Informatics Innovations for Sustainable Health Care Systems. (2024). p. 157–8. doi: 10.3233/SHTI240367 39176696

[69] Alkarkoukly S Kamal MM Beyan O. Breaking Barriers for Interoperability: A Reference Implementation of CSV-FHIR Transformation Using Open-Source Tools. Amsterdam: IOS Press (2023). doi: 10.3233/SHTI230061 37203606

[70] ITF for Education. IT Academy Research Support Measures Programme for 2018-2022: Artificial Intelligence & Machine Learning; Data Science and Big Data; Robots-People collaboration and the Internet of Things in Industry Processes. (2018). Available online at: https://www.etis.ee/Portal/Projects/Display/5c855a0c-74e9-4af3-9cc9-5b13c4cd6e0b (Accessed May 23, 2024).

[71] Estonian Research Council. Digital Health for a Whole and Healthy Society, 2024-2028. (2024). Available online at: https://health.ec.europa.eu/publications/proposal-regulation-european-health-data-space_en (Accessed February 01, 2025).

[72] Estonian Research Council. Medication Adherence and Treatment Efficacy in Patients with Dyslipidaemia and Achievement-Oriented Novel Patient Digital Support, 2025-2029. (2025).

[73] Ardel H. K. (2025). Enabling the transformation of health data between HL7 FHIR and OMOP CDM (Master's thesis). Available online at: https://digikogu.taltech.ee/et/Item/ca6d6a65-c97e-451b-8024-24941538c9e3 (Accessed January 27, 2026).

